# Nrf2–Keap1 Axis Modulation by Phytofabricated Metal Oxide Nanoparticles in Mesenchymal Stem Cell Lineage Bias

**DOI:** 10.1002/jbt.71091

**Published:** 2026-08-31

**Authors:** Muhammad Irfan, Muhammad Taha, Syed Muhammad Faizan Asghar, Muhammad Raza

**Affiliations:** ^1^ Department of Biochemistry and Biotechnology University of Gujrat Gujrat Pakistan; ^2^ Institute of Veterinary Medicine and Animal Husbandry, Chair of Veterinary Biomedicine and Food Hygiene Estonian University of Life Sciences Estonia

**Keywords:** bone regeneration, mesenchymal stem cells (MSCs), Nrf2–Keap1 signaling, osteogenic differentiation, redox homeostasis

## Abstract

Mesenchymal stem cells (MSCs) hold considerable therapeutic promise in regenerative medicine, yet precise control over lineage fate remains critical for reproducible clinical outcomes. This review examines the Nrf2–Keap1 (nuclear factor erythroid 2–related factor 2–Kelch‐like ECH‐associated protein 1) signaling axis as a redox‐sensitive rheostat governing the balance between osteogenic and adipogenic differentiation. Physiological reactive oxygen species (ROS) support osteogenesis in a dose‐ and context‐dependent manner, whereas sustained oxidative stress drives cellular senescence and adipogenic bias; emerging evidence also indicates that Nrf2 hyperactivation can itself impair osteogenic differentiation, pointing to an optimal activation window rather than a simple “more is better” relationship. Conventional plant‐derived Nrf2 activators are limited by poor bioavailability and metabolic stability. Phytofabricated metal oxide nanoparticles (MONPs), synthesized using plant extracts as capping and stabilizing agents, offer a biocompatible, “green” delivery alternative. We propose that these hybrid systems may act through a dual‐stimulus mechanism — phytochemical‐mediated electrophilic modification of Keap1 cysteines alongside metal‐oxide‐core‐derived, sub‐cytotoxic ROS consistent with mitohormetic signaling — that together could promote Nrf2 nuclear translocation, ARE‐driven antioxidant gene expression, and downstream epigenetic reinforcement of osteogenic commitment. This dual‐stimulus framework, however, remains a mechanistic hypothesis: no study to date has directly demonstrated Keap1 modification, Nrf2 activation, and resulting lineage bias by phytofabricated MONPs within MSCs. Preclinical studies support the efficacy of phytofabricated MONPs in bone defect models and suggest potential for restoring function in senescent MSCs, but direct mechanistic validation in MSC systems remains an essential next step before these insights can guide rational nanoplatform design for bone regeneration.

## Graphical abstract

Phytofabricated metal oxide nanoparticles may activate the Nrf2–Keap1 axis through a dual‐stimulus mechanism, coupling nanoparticle‐derived ROS with phytochemical‐mediated Keap1 modification, steering mesenchymal stem cells toward osteogenic commitment — a mechanistic framework this review consolidates from current evidence.

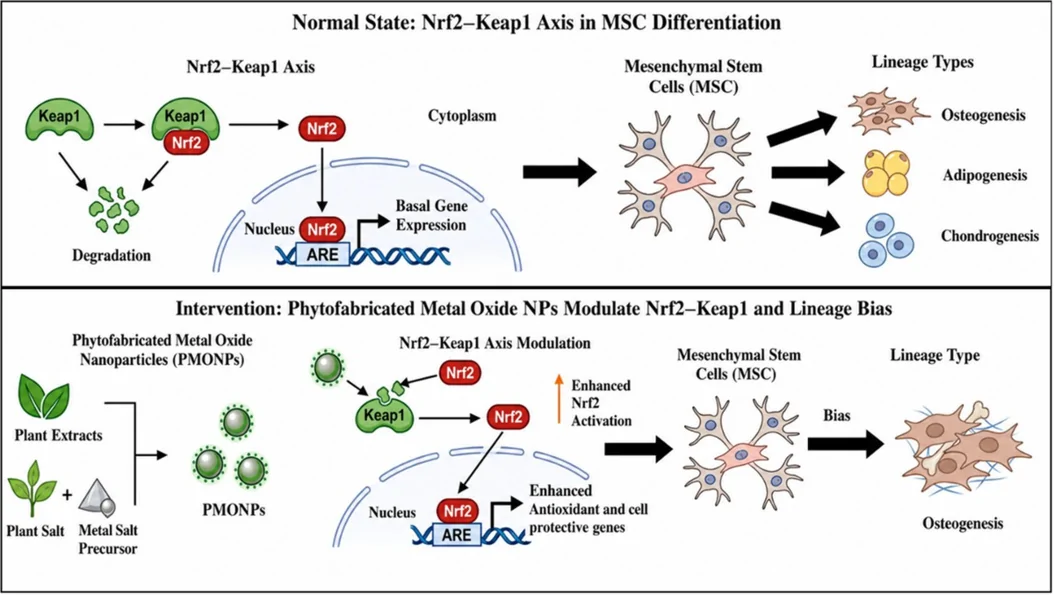

## 1 Introduction

Mesenchymal stem cells (MSCs) are multipotent adult cells that can self‐renew and differentiate into various mesenchymal cell types, such as osteoblasts, chondrocytes, and adipocytes. MSCs were originally identified in bone marrow, but are now also isolated from adipose tissue, dental pulp, and umbilical cord tissue, among other sources. The anatomical origin of MSCs seems to be intrinsic to their lineage potential; for instance, the bone marrow‐derived MSCs have an osteogenic bias, while those of adipose tissue are generally adipogenically biased [1]. The study of MSCs is prevalent in the clinical trials performed in the field of regenerative medicine because of their ability to localize in various tissues, immunomodulatory actions, and multiple lineage capacity. But to achieve a reproducible therapeutic outcome, their lineage commitment must be carefully controlled [2].

Commitment to MSC lineage is regulated by biochemical, mechanical, and cytoskeletal signals, such as substrate stiffness and cell morphology/motility, that are transmitted to the cell interior via pathways such as RhoA/ROCK and YAP/TAZ [3]. Along with these biomechanical inputs, the intracellular redox state, which is primarily controlled by mitochondrial reactive oxygen species (ROS), is a crucial factor in MSC fate. ROS have a biphasic regulatory action – physiological levels act as secondary messengers, promoting proliferation/differentiation, while high levels contribute to oxidative stress, cellular senescence and abnormal lineage development. Predictable differentiation of MSCs is thus a prerequisite for redox balance [4]. Recent studies suggest that this redox‐regulated modulation of stem cell fate is controlled by a signaling pathway that involves the nuclear factor erythroid 2‐related factor 2 (Nrf2)–Kelch‐like ECH‐associated protein 1 (Keap1) [5].

Cells have an inducible cytoprotective defense mechanism that protects them against stress‐induced damage that is coordinated by the Nrf2–Keap1 pathway, among others [6]. In the absence of stress, Nrf2 is retained in the cytoplasm by binding with Keap1, which promotes its degradation by the proteasome [7]. Under oxidative or electrophilic stress, Keap1 releases Nrf2 which translocates to the nucleus and activates a wide range of cytoprotective anti‐oxidant response element (ARE)–controlled genes [8]. This dynamic regulation allows the Nrf2–Keap1 axis to act as a key sensor and effector of redox homeostasis and adaptive responses to stress [7].

The Nrf2–Keap1 axis is known to be a crucial pathway in the regulation of cell specification in MSCs. This pathway has been found to fine‐tune cell fate decisions, preventing pathological trends toward adipogenesis in pro‐inflammatory or oxidatively stressed microenvironments, and retaining osteogenic potential in MSCs; whether the calibration is causal or correlative is an ongoing topic of investigation in this field [9]. This axis is thus a rational target to guiding the allocation of the MSC lineage in therapy. Bioactive natural compounds derived from plants (polyphenols, flavonoids, terpenoids) directly activate Nrf2, but their poor cell uptake, rapid metabolism and lack of targeted delivery limits their application as activators in the field of stem cell therapy [10]. In order to overcome these limitations, delivery methods based on nanotechnology have been gaining interest and phytofabricated metal oxide nanoparticles (MONPs) have been found as a comparatively stable delivery platform [11].

Phytofabricated MONPs are those that are fabricated with the help of plant extracts where secondary metabolites like polyphenols, flavonoids, and terpenoids act as reducing, capping, and stabilizing agents while fabricating the particles [12, 13]. Compared to the traditional synthesis techniques, which demand toxic chemical reactants that lead to toxic by‐products, the phytofabrication method provides an eco‐friendly approach for the synthesis of biocompatible nanoparticles with tunable physico‐chemical properties and low toxic by‐products. In addition to being passive carriers, the corona of phytochemicals that remains on the NPs might have other antioxidant and anti‐inflammatory properties. Due to the incorporation of bioactive molecules within the nanoscale constructs, phytofabrication could be a promising alternative to the limitations in delivering isolated phytochemicals [14].

We hypothesize that the internalized phytofabricated MONPs can modulate the Nrf2–Keap1 axis in MSCs via a two‐stimulus effect: (1) ROS signaling from the metal oxide core that mimics mitohormetic signaling and (2) electrophilic modification of the Keap1 cysteine residues by the phyto‐chemical corona [15]. These other stimuli may stabilize, translocate, and activate Nrf2 via ARE‐mediated transcription [15, 16]. It must be emphasized that this dual‐stimulus framework is a mechanistic hypothesis synthesized largely from evidence in non‐MSC systems; direct experimental demonstration of Keap1 modification, Nrf2 activation, and consequent lineage bias by phytofabricated MONPs within MSCs themselves is currently lacking, and this distinction is maintained throughout the review.

In spite of increasing evidence on the redox‐modulating properties of phytofabricated MONPs, there are no such direct reports of modulation of Nrf2–Keap1 pathway by such nanomaterials in MSCs or the changes in MSC lineage bias [17]. This knowledge gap is interconnected with the fields of MSC redox biology, nanoformulation of phytochemicals and green nanotechnology and has direct implications for the rational design of nanoplatforms for various use cases where reproducible osteogenic commitment is required, such as bone regeneration. This narrative review therefore attempts to (i) summarize existing evidence for the redox‐modulatory and lineage‐directing effects of phytofabricated Nrf2–Keap1 modulation, (ii) critically evaluate evidence for the role of Nrf2–Keap1 modulation in other cell types but also in MSCs, with explicit distinction between direct evidence specific to the MSC and evidence extrapolated from other cell types, and (iii) propose a mechanistic framework that is clearly formulated as a hypothesis‐generating model, based on cysteine modification of Keap1, Nrf2 transcriptional activation and suppression of adipogenic regulators. In a section later in this review, the following issues that may be of toxicological or biosafety concerns for translational use are discussed.

### 1.1 Methodology

**Figure 1:**
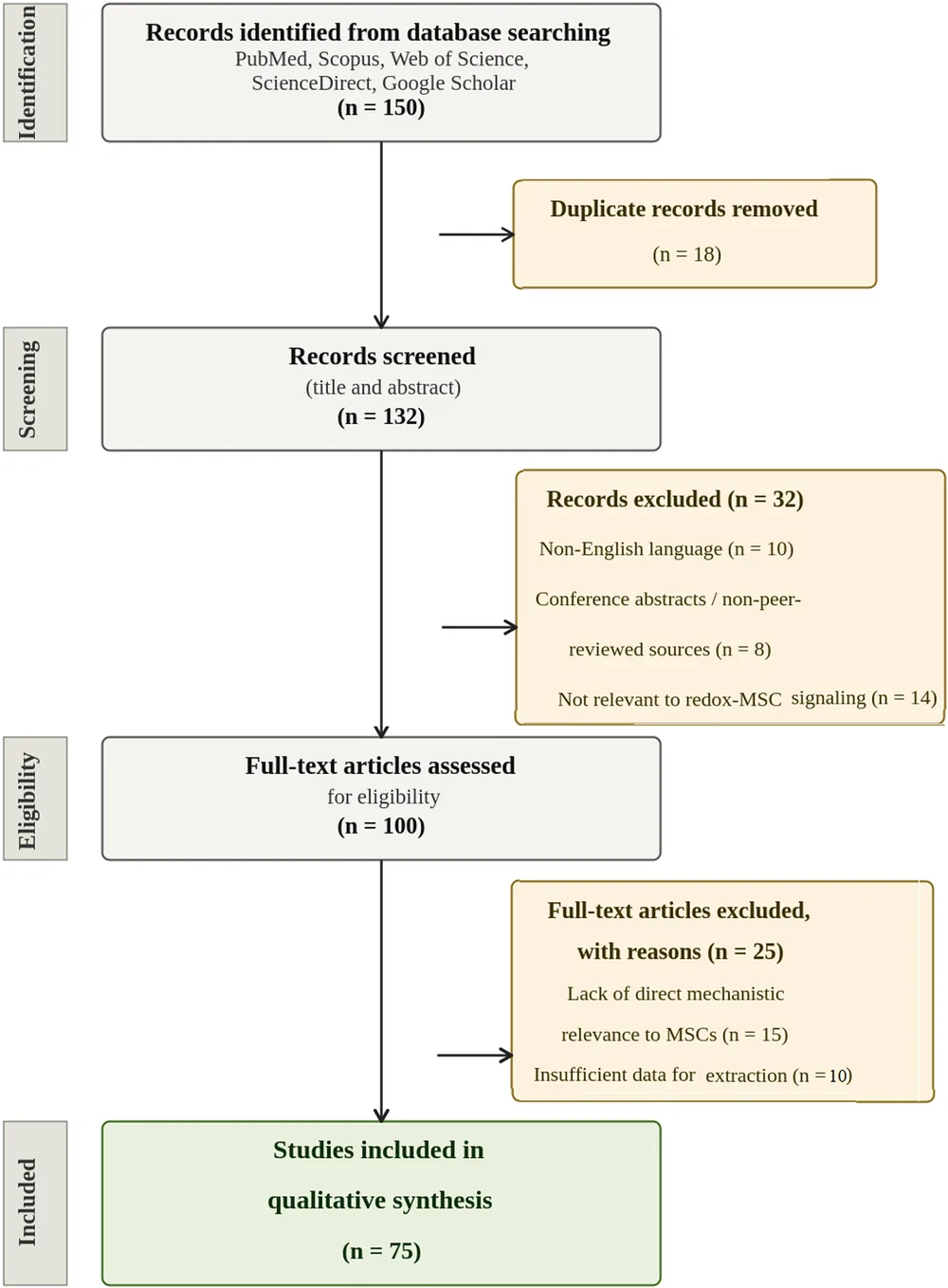
PRISMA flow diagram for the selection of studies.

This review was conducted as a narrative synthesis, supported by a systematic search strategy that include, but was not limited to, evidence of redox biology, stem cell research, nanotechnology and phytochemistry. The available evidence was synthesized to evaluate the role of phytofabricated MONPs in regulating the Nrf2–Keap1 axis and MSC lineage commitment. Relevant research, mainly published between 2020 and 2026 was located from PubMed, Scopus, Web of Science, ScienceDirect, and Google Scholar using various combinations of keywords. Studies published in English, peer‐reviewed, in vitro, in vivo or in silico (e.g., molecular docking), which provided mechanistic insight into Nrf2 activation, and into redox‐dependent MSC differentiation or MONP bioactivity were included, while non‐English publications, conference abstracts and mechanistically irrelevant studies were excluded. Records were reviewed using title/abstract selection, and subsequently by full‐text review to determine eligibility, resulting in 75 studies included (Figure 1, PRISMA‐style flow diagram). Resulting data were organized into pathway architecture, redox‐lineage regulation, NPs synthesis/characterization, dual‐stimulus activation mechanisms, and downstream transcriptional effects, with computational or non‐MSC‐derived evidence flagged accordingly. Figures were prepared using BioRender, Canva, and PowerPoint.

## 2 Molecular Architecture of the Nrf2–Keap1 Signaling Axis

### 2.1 Structural Organization of Nrf2 and Keap1

Much of the regulatory control of Nrf2 function in the Keap1 signaling axis is through the molecular architecture of these two components. Nrf2 is part of the Cap'n'collar (CNC) family of basic leucine zipper (bZIP) transcription factors and has six functionally distinct Neh (Nrf2‐ECH homology) domains. The Neh1 domain contains the bZIP/CNC DNA‐binding region that needs to heterodimerize with small Maf proteins to bind antioxidant response elements (AREs) [17]. The Neh2 domain is the principal Keap1‐binding site, which comprises two degron motifs: high‐affinity ETGE motif and lower‐affinity DLG motif, whose differential binding to Keap1 accounts for the “hinge‐and‐latch” model of Nrf2 sequestration [6].

### 2.2 Basal Ubiquitination and Continuous Proteasomal Turnover

In the absence of stress, Nrf2 is constitutively degraded by ubiquitin proteasomes in a sustained manner, which allows for the rapid response to redox stress [6]. The asymmetric bivalent binding of each Nrf2 molecule to a single Keap1 homodimer is mediated by the high affinity ETGE binding motif in one Kelch propeller and the low affinity DLG binding motif in the other [18]. This dual occupancy correctly positions certain lysine residues (K44, K50, K52, K53) in the Nrf2 Neh2 domain in relation to the ubiquitin ligase complex Keap1–Cullin3–RBX1. The Cullin3 scaffold links Keap1 to the RING‐domain protein RBX1, which directs an E2 enzyme to add ubiquitin to these lysines [6, 18]. The basal half‐life of Nrf2 is about 10–30 min due to the rapid degradation of poly‐ubiquitinated Nrf2 by the 26S proteasome. The half‐life of the Nrf2 protein is short, which depends mainly on the rate at which Nrf2 escapes degradation, thereby enabling rapid, reversible responsiveness to the transient redox changes that occur in MSC niches [7].

### 2.3 Redox‐Sensitive Cysteine Sensors in Keap1: The Molecular Switch

**Figure 2:**
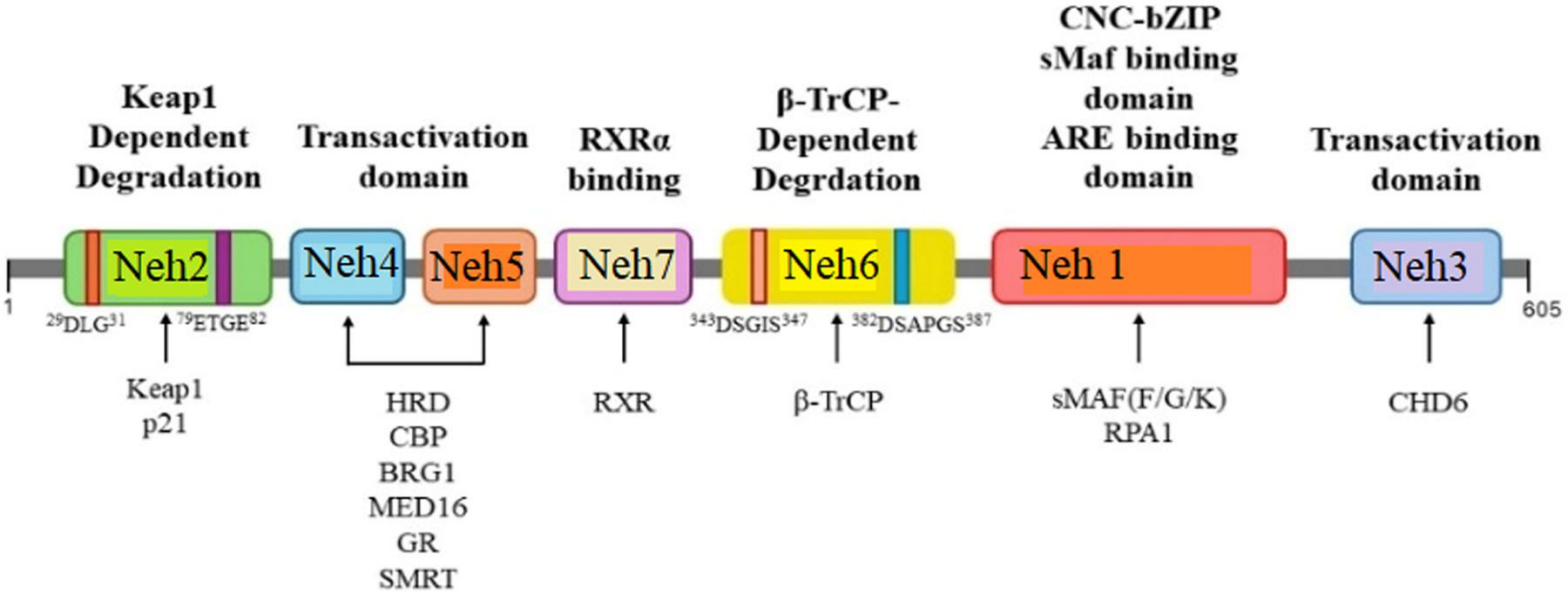
Linear domain maps of Nrf2 (Neh1–6, with ETGE/DLG degrons) and Keap1 (NTR, BTB, IVR, Kelch/DGR, CTR) with reactive cysteines (Cys151, Cys273, Cys288, Cys434, Cys489) marked. (Made in PowerPoint) [6]. Of its 27 cysteine residues, three are established as principal sensors. The BTB‐domain Cys151 acts as an electrophilic sensor, modified directly by α, β‐unsaturated carbonyls and isothiocyanates; this disrupts the BTB–Cullin3 interaction, impairs E3 ligase assembly, and prevents Nrf2 ubiquitination. Cys273 and Cys288, located in the intervening region (IVR), function primarily as ROS sensors [19].

Keap1 converts chemically diverse stress signals into defined conformational responses through a combinatorial cysteine‐based sensing mechanism (Figure 2).

### 2.4 Downstream Transcriptional Network and Pathway Crosstalk

After being released from Keap1‐dependent repression, Nrf2 translocates to the nucleus where it binds small Maf proteins (MafG, MafF, MafK) via its Neh1 bZIP domain. This complex binds specifically to ARE consensus sequences (5'‐TGACnnnGC‐3'), which coordinately promote expression of a broad cytoprotective gene program [6] which includes heme oxygenase‐1 (HO‐1), NAD(P)H quinone oxidoreductase 1 (NQO1), glutamate‐cysteine ligase subunits (GCLC/GCLM), thioredoxin reductase 1 (TXNRD1), superoxide dismutases (SOD1/SOD2), and ferritin [7]. In addition to the pattern of Keap1 cysteine modification, small Maf protein composition induces a further degree of transcriptional selectivity, which influences the pattern of gene expression in response to various stimuli. The Nrf2–Keap1 axis is involved in several regulatory pathways in addition to its fundamental role in the antioxidant program. Interestingly, Nrf2 suppresses NF‐κB activation in the expression of pro‐inflammatory genes by competing with NF‐κB for co‐activators like CBP/p300 and by inhibiting HO‐1 production of CO [20] that has direct relevance to inflammatory bone loss in the osteogenic niche. Nrf2 also interacts with the FOXO family that regulates longevity and resistance to stress in cells, and p53, a stress‐responsive protein that determines the survival or death of cells. These crosstalk systems have indirect but important connections to the specification of MSC fate, which are discussed in Sections 3 and 6 when considering osteogenic–adipogenic lineage specification and the signaling activity of phytofabricated MONPs, respectively [21].

## 3 Nrf2–Keap1 Regulation of Mesenchymal Stem Cell Lineage Commitment

### 3.1 Nrf2 in Osteogenic Differentiation

The MSC redox environment is not a neutral environment that simply accompanies lineage commitment, but is an active modulator of the transcriptional fate [22]. Mitochondrial ROS seems to exert a biphasic effect on osteogenic differentiation; physiological ROS levels serve as permissive second messengers that activate pro‐osteogenic kinase cascades, whereas super‐physiological ROS levels inhibit osteogenesis and lead to oxidative harm [23]. Under these conditions, the Nrf2–Keap1 axis is a major regulator of intracellular ROS in this permissive range, as Nrf2 will induce antioxidant and cytoprotective enzymes, helping to prevent a shift from instructive ROS signaling to pathological oxidative stress that promotes continued osteoblast differentiation [9].

### 3.2 The Dual/Context‐Dependent Role of Nrf2 in Osteogenesis

There is no consistent positive relationship between Nrf2 activity and osteogenic differentiation; this is a point that should be clearly acknowledged. Under certain conditions, such as a challenge of oxidative stress, moderate Nrf2 activity seems to support osteoblast cytoprotection (Section 3.1), but some studies indicate that hyperactivation or overexpression of Nrf2 may negatively affect osteogenic differentiation. It was demonstrated that overexpression of Nrf2 in murine MC3T3‐E1 osteoblasts inhibited the transactivation of the osteocalcin promoter by Runx2, which did not occur through changes in Runx2 mRNA levels [24]. More directly pertinent to this review, a recent study in human bone marrow‐derived mesenchymal stromal cells found that pharmacologic activation of Nrf2 reduced the expression levels of RUNX2‐targeted genes (such as ALP) to inhibit osteogenic differentiation, while simultaneously increasing mineralization [25]. The relevance of this work in mouse models is also that it suggests that Nrf2 is a context‐dependent regulator of bone acquisition, not a simple pro‐osteogenic factor [26]. In summary, these results indicate an optimal‐window model: insufficient Nrf2 activity to prevent oxidative damage, but excessive activity to directly antagonize RUNX2‐dependent transcription, which suggests that the therapeutic window for phytofabricated MONP‐based Nrf2 modulation is titrated activation and not maximal activation.

### 3.3 Nrf2 in Adipogenic Commitment and Its Suppression

Unlike its role in osteogenesis, it has been demonstrated that activation of Nrf2 leads to a suppressive effect on adipogenic differentiation, suggesting that the Nrf2–Keap1 axis is a redox‐sensitive rheostat that regulates the osteogenic–adipogenic balance (Table 1) [5]. Transcriptional activation of CCAAT/enhancer‐binding protein alpha (C/EBPα) followed by peroxisome proliferator‐activated receptor gamma (PPARγ), the master adipogenic regulator, is required for adipogenesis. Interestingly, oxidative stress stimulates both pathways that promote adipogenesis, in spite of their opposing effects: ROS increases the expression of C/EBPα by transactivating AP‐1, and ROS increases the stability of both the mRNA and protein of PPARγ in the presence of chronic ROS, mediated by the activation of p38 MAPK [5]. Endogenous electrophilic PPARγ activators such as 4‐hydroxynonenal (4‐HNE) and 15‐deoxy‐Δ12,14‐prostaglandin J_2_ (15d‐PGJ_2_) are both products of lipid peroxidation under high ROS levels and directly couple oxidative stress to adipogenic transcription by covalently modifying a ligand‐binding domain cysteine (Cys285) [6]. Importantly, these same electrophiles can also modify Keap1 cysteines (Cys273, Cys288), which sets up a situation where endogenous, lipid‐derived electrophiles can concurrently activate PPARγ and Nrf2; signaling outcomes in the lineage will depend on the relative intensity and timing of each modification. This is only a proposed mechanistic model and not yet proven to be a direct pathway in MSCs, marked as such [30].

**Table 1:** Nrf2 signaling in MSC lineage‐specific differentiation.

Lineage	Key TFs and markers	Nrf2 status and role	Key ARE targets	Functional outcomes	Reference
Osteogenic	RUNX2 (Rust‐ related transcription factor), SP7, DLX5, ATF4 (Activating transcription factor 4)	Active: Preserves osteoblast viability and RUNX2 function against oxidative inactivation.	HMOX1, NQO1, GCLC/M, TXNRD1, SOD1/2, FTH1/FTL, SRXN1.	ALP, BGLAP, SPP1, Col I, mineralization, RUNX2 oxidation.	[27]
Adipogenic	PPARγ(Peroxisome Proliferator‐Activated Receptor Gamma, C/EBPα/β, SREBP1c Sterol Regulatory Element‐ Binding Protein 1c	Suppressed: Degradation allows ROS/lipids to drive PPARγ. Nrf2 reactivation inhibits PPARγ via competition.	Suppressed (e.g., HMOX1, GCLC, NQO1, GPX4, (Glutathione Peroxidase 4).	Lipids, FABP4, adipokines, ALP, RUNX2 (Nrf2 activation reverses this).	[28]
Chondrogenic	SOX9, HIF‐1α, ACAN(Aggrecan), COL2A1, COMP(Cartilage Oligomeric Matrix Protein)	Moderately active: Cytoprotective in hypoxic niche; buffers ROS alongside HIF‐1α.	SOD2, TXNRD1, PRDX3, GPX1/4, HMOX1, CAT, GCLC.	Col II, proteoglycans; Col X, MMP‐13, ADAMTS5 (Prevents hypertrophy).	[29]

### 3.4 Nrf2 and MSC Senescence: Redox Erosion of Regenerative Potential

A dimension of Nrf2–Keap1 biology in MSCs that has gained increasing mechanistic clarity is the pathway's direct involvement in the regulation of cellular senescence and the progressive erosion of regenerative capacity with aging [21]. Cellular senescence in MSCs is characterized by irreversible growth arrest mediated through the p53–p21 and p16INK4a–retinoblastoma protein axes, activated in response to persistent ROS‐driven DNA damage, telomere attrition, and chronic mitochondrial dysfunction. As MSCs undergo replicative or stress‐induced senescence, there is a well‐documented decline in Nrf2 nuclear activity that is attributable to multiple converging mechanisms: increased Keap1 protein expression driven by senescence‐associated epigenetic changes, elevated GSK‐3β activity that promotes the β‐TrCP‐mediated Neh6 domain degradation of Nrf2, and the accumulation of oxidatively damaged proteins that sequester chaperones required for efficient Nrf2 nuclear import [31]. This senescence‐associated Nrf2 insufficiency creates a feed‐forward oxidative cycle in which declining antioxidant capacity permits further ROS accumulation, additional DNA damage, and deepening senescence [21].

Evidence that Nrf2 reactivation reduces SASP secretion, decreases p21 and p16 expression, and partially restores osteogenic commitment in aged MSC populations provides proof of concept for this therapeutic rationale and establishes a clear experimental precedent for evaluating the anti‐senescence potential of phytofabricated MONPs in aged MSC systems, a translational application addressed directly in Section 10.2 [21, 31]. (same as original).

## 4 Phytofabricated Metal Oxide Nanoparticles: Green Synthesis and Physicochemical Properties

### 4.1 Principles and Mechanistic Basis of Phytofabrication

Phytofabrication utilizes the reducing, chelating, and stabilizing properties of the secondary metabolites produced in the plant to facilitate the controlled nucleation and growth of metal oxide nanocrystals from the dissolved metal salt precursors in the mild aqueous conditions, eliminating the need for toxic reducing agents such as sodium borohydride, hydrazine hydrate, and dimethylformamide, which are used in conventional synthesis processes [32]. These constructs are not just “green” alternatives to chemically synthesized nanoparticles, but are actually compositionally different hybrid nano‐bio systems whose surface chemistry is tailored by the specific phytochemicals that are recruited during their bio‐fabrication, and that bring the properties of biocompatibility, sustainability, and functional surface activity – all in one bio‐fabrication step [15].

**Figure 3:**
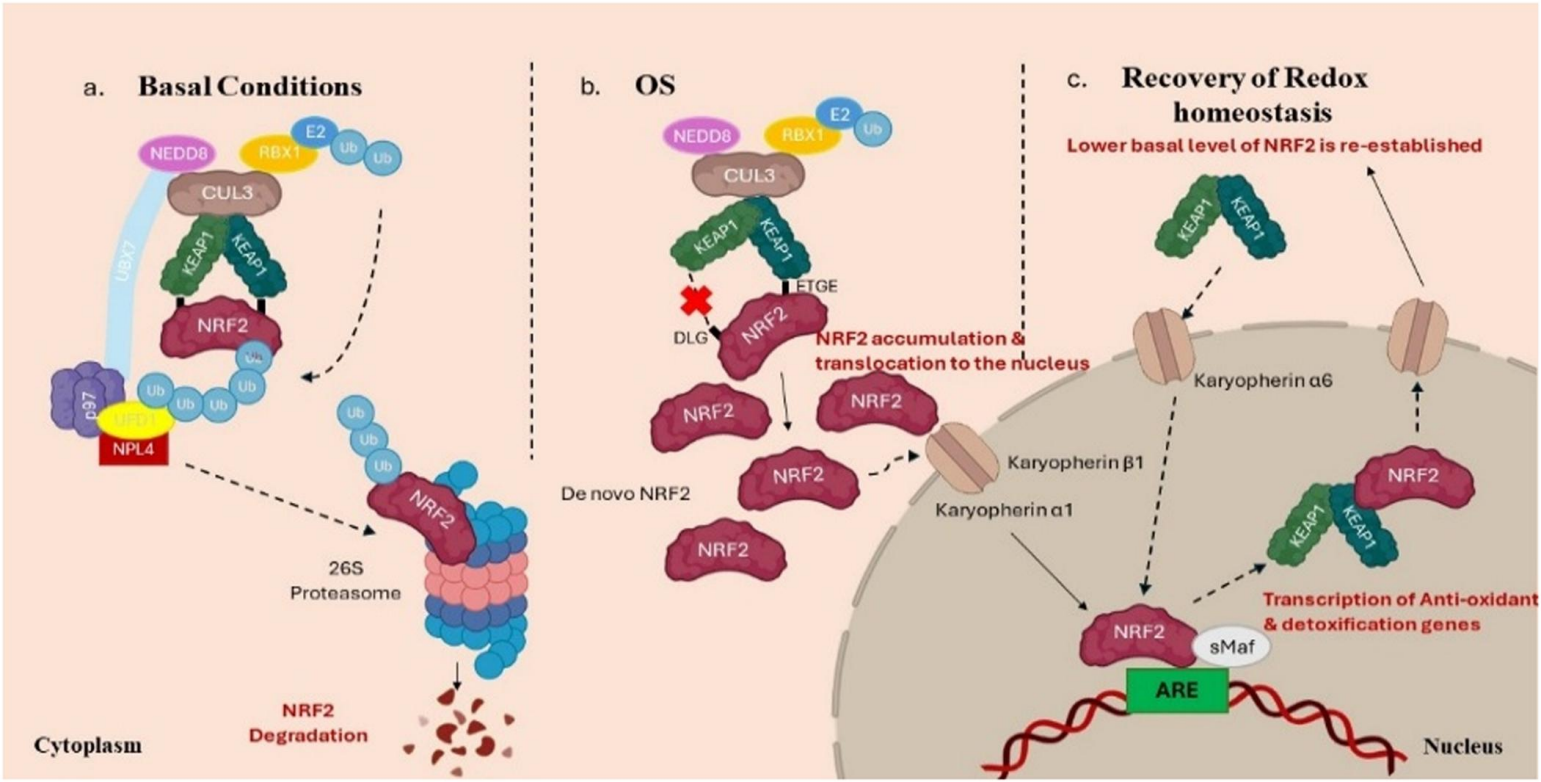
A dynamic mechanistic cycle contrasting basal conditions—showing the Keap1–Cullin3–RBX1 complex driving continuous Nrf2 degradation—with the stress state, in which cysteine modification disrupts Keap1 adapter function, permitting Nrf2 stabilization. (a) Basal conditions: Keap1 recruits Nrf2 to the Cul3–RBX1 E3 ubiquitin ligase complex, promoting its ubiquitination and subsequent degradation by the 26S proteasome. (b) Oxidative stress (OS): Modification of critical Keap1 cysteine residues impairs Nrf2 ubiquitination, resulting in Nrf2 stabilization, accumulation, and translocation into the nucleus. (c) Recovery of redox homeostasis: Nuclear Nrf2 binds antioxidant response elements (AREs) and induces antioxidant and detoxification genes, restoring cellular redox balance and reducing Nrf2 activity toward basal levels. (Made using Biorender & PowerPoint).

The major phytochemical classes play mechanistically different roles [33]. Polyphenols such as gallic acid, ellagic acid, and EGCG donate electrons from the hydroxyl groups to reduce the metal cations (Zn^2+^, Ti^4+^, Fe^3+^, Ce^4+^) and initiate nucleation at around 60°C–80°C; the planar aromatic ring structures coordinate interactions at the surface of the nascent nanocrystals. Terpenoids are involved in the assembly of the corona with hydrophobic and hydrogen‐bonding interactions, which passivate surface defects and give corona stability over a long time (Figure 3) [32]. The Corona of these phytochemicals is therefore chemically bound during their synthesis instead of just being electrically adsorbed later, and for this reason, the surface functionalization cannot be reproduced by post‐synthetic grafting [33].

### 4.2 Clinically and Mechanistically Relevant Metal Oxide Nanoparticles

Here, we discuss four metal oxide systems that have enough evidence in stem and progenitor cell systems to warrant detailed consideration: each shows different redox chemistry and an associated unique hormetic profile when paired with its phytochemical corona [34].

Phytofabricated zinc oxide (ZnO) nanoparticles, using extracts like *Aloe vera*, *Azadirachta indica*, and *Moringa oleifera*, produce intracellular ROS by the endolysosomal dissolution, releasing zinc ions (Zn^2^+), which disrupt the activity of mitochondrial Complex I and III, as well as by the formation of hydroxyl radicals on the surfaces of these nanoparticles. This dual ROS source induces an osteogenic hormetic dose of Nrf2 activation and also simultaneously stimulates Wnt/β‐catenin and BMP/Smad signaling, which occurs in parallel with Nrf2 [34]. UV exposure will cause the photocatalytic generation of ROS by TiO_2_ nanoparticles, while physiologic conditions produce a comparatively lower/moderate ROS, and phytofabrication will shift their optical absorption toward the visible spectrum, increasing the density of surface hydroxyl groups, which will help improve colloidal stability and allow for more tunable redox control [35].

### 4.3 The Phytochemical Corona: Surface Chemistry and Retained Bioactivity

FTIR spectra of phytofabricated MONPs consistently show the presence of hydroxyl (3200–3500 cm^−1^), carbonyl (1620–1720 cm^−1^), carboxylate (1380–1540 cm^−1^), and amine (1530–1650 cm^−1^) functional groups, suggesting surface retention of secondary metabolites. The resultant zeta potentials (around −20 to −45 mV) revealed electrostatic colloidal stability at a physiological ionic strength and enabled reproducible cell dosimetry and intracellular trafficking [36].

The question of whether the electrophilic corona‐bound phytochemicals will retain their electrophile reactivity after release in the cell to create covalent adducts with the cysteines of Keap1 remains a mechanistic issue of interest [36]. The interaction of surface‐bound polyphenols with metal oxide surfaces is thought to occur mainly via the catechol/galloyl hydroxyl groups, which are able to be oxidized to electrophilic quinone‐type species once the corona is disassembled, ready for nucleophilic attack by the Keap1 thiol groups [37]. As mentioned above, the model of the multiple interaction sites for Keap1 with the right partner involves Michael addition of surface‐released curcuminoids to Cys151 and complementary oxidative modification of Cys273/Cys288 by surface‐bound quercetin/EGCG under transition‐metal‐catalyzed conditions [38]. The corona‐reactivity claims are now based on spectroscopic and modeling inferences, and the claims should be interpreted as a possible mode of action until the formation of the biochemical adduct has been confirmed by mass spectrometry in cells.

### 4.4 Redox‐Active Nanozyme Properties

Surface redox valence distribution, particle size, and local pH control the relative SOD‐mimetic, catalase‐mimetic, and peroxidase‐mimetic activities of CeO_2_ and Fe_3_O_4_ NPs (Table 2) [38]. CeO_2_ nanoparticles catalytically eliminate superoxide and hydrogen peroxide while Fe_3_O_4_ nanoparticles produce hydroxyl radical as part of a Fenton‐type peroxidase‐like chemistry — reactions with opposing mechanisms which directly define the hormetic Nrf2‐activation profile of a particular nanoparticle system [41].

**Table 2:** Physicochemical features of phytofabricated MONPs related to MSC applications.

NP type	Key plant sources	ROS modulation profile	Bio effects in stem cells	Reference
ZnO	*Aloe vera, Moringa, Camellia sinensis*.	Pro‐oxidant (dual at high dose), Zn^2+^ drives ROS: corona buffers toxicity, hormetic at the 1–25 µg/mL.	ALP, RUNX2, mineralization. Nrf2 (HO‐1/NQO1): senescence/NF‐κB(Nuclear Factor Kappa B).	[39]
TiO_2_	*Azadirachta, Curcuma, Ocimum*	UV‐dependent pro‐oxidant (•OH/O_2_•^−^), corona enhances surface hydroxyls and buffers high‐dose ROS.	ALP(Alkaline Phosphatase), Col I, OCN; osteoblast adhesion; Nrf2 targets; inflammation.	[40]
Fe_3_O_4_	*Punica, Rosmarinus, Urtica*	Dual: Fenton reaction (Fe^2+^) pro‐oxidant versus corona antioxidant: antioxidant dominant ≤ 10 µg/mL.	RUNX2, ALP(Alkaline Phosphatase), Nrf2 via p62‐Keap1 autophagy, magnetic targeting enabled.	[39]
CeO_2_	*Acacia, Olea, Hibiscus*	Strong antioxidant (nanozyme): Ce3+↔Ce4+ cycling scavenges ROS, indirectly activating Nrf2.	ALP, RUNX2, Nrf2 (HO‐1/SOD2): strong cytoprotection, SASP/adipogenesis.	[30]

## 5 Computational Evidence of Keap1 Phytochemical Interactions

### 5.1 Rationale for in Silico Approaches in Nrf2 Activator Screening

In silico methods represent a relatively efficient screening of potential Nrf2 activators compared to empirical methods [42]. The structural diversity of phytochemicals and the number of mechanistically different sites of interaction with Keap1, by which different molecules can act (competitive ETGE‐pocket occupancy *vs.* covalent modification of cysteine), make the identification of molecules capable of disrupting the Keap1–Nrf2 association a non‐trivial screening problem. In contrast, empirical cell‐based screening is relatively expensive, may be complicated by cytotoxicity and/or off‐target redox signaling, and offers little mechanistic insight into the mechanism responsible for Nrf2 stabilization [43].

### 5.2 Molecular Docking, Phytochemical Ligands With Keap1 Domains

**Figure 4:**
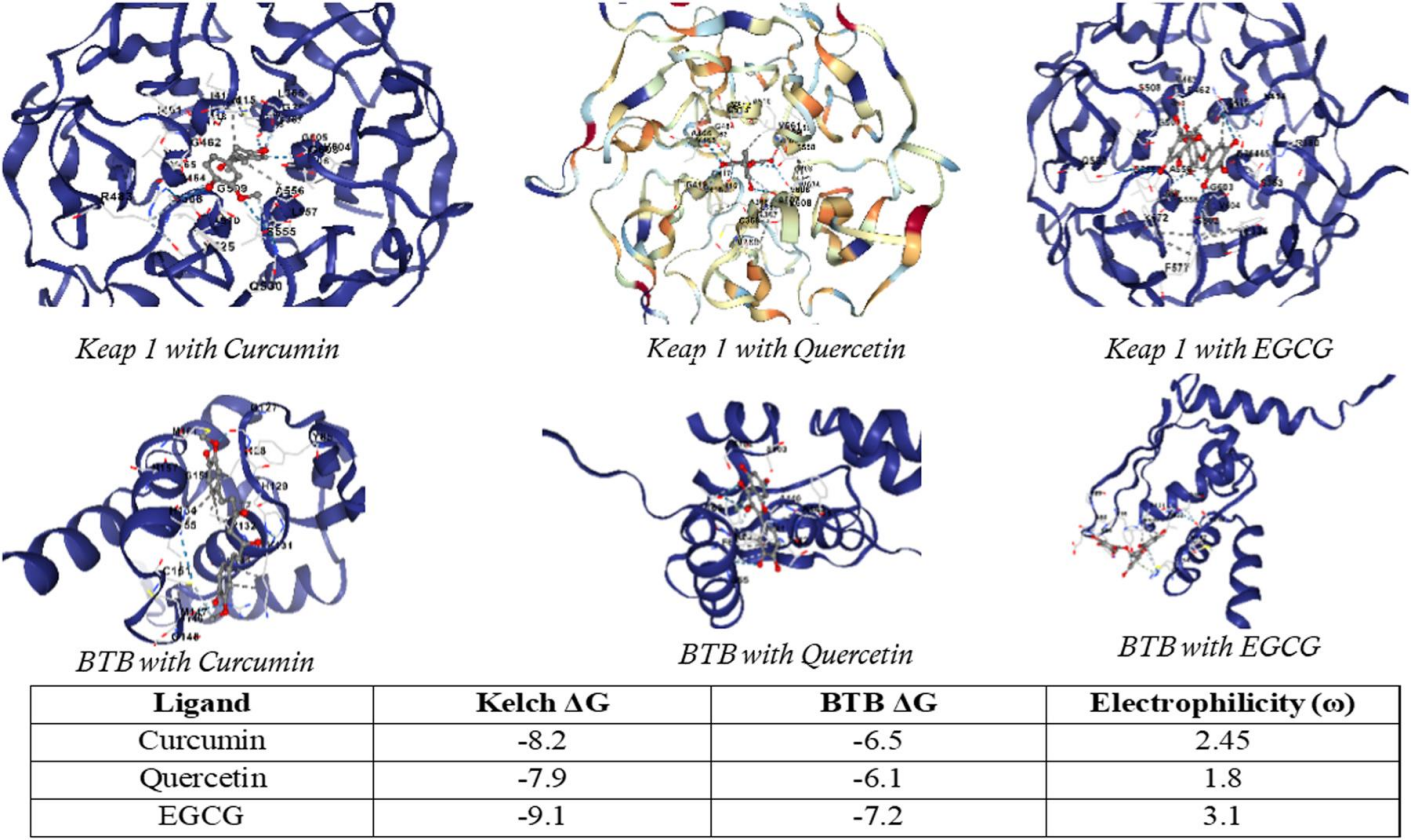
Dual‐site docking analysis of curcumin, quercetin, and EGCG at the Keap1 Kelch and BTB domains, featuring electrostatic surface mapping and key residue interactions. The graphic includes a comparative chart of binding affinities relative to the native ETGE peptide and calculated electrophilicity indices.

The computational docking studies of common dietary phytochemicals show evidence of binding to Keap1 at non‐covalent and covalent binding sites [42]. The free energies of binding of curcumin to the Kelch‐domain were favorably negative, ranging from −8 to −10 kcal/mol, with hydrogen bond interactions between the diketone and hydroxyl groups of curcumin and the Kelch‐domain residues Arg415, Arg483, Ser508, and Tyr572 that are important anchoring residues for the ETGE motif of Nrf2 (Figure 4) [42, 43]. Such binding geometry positions the α, β‐unsaturated carbonyl group of curcumin very close to the BTB Cys151 pocket, suggesting a dual binding mode of non‐covalent binding to the BTB and covalent Michael adduct formation [42]. Quercetin and kaempferol exhibit similar Kelch‐domain affinities (−7.8 to −9.4 kcal/mol), which reproduce critical arginine/serine interactions in the ETGE‐binding groove through their catechol hydroxyl groups. EGCG's gallate ester has also been suggested to directly bind to and modify Tyr334, which lacks the ability to bind to the same residue in simpler flavonoid structures, and could account for its relatively high potency in activating the Nrf2 pathway within cells; however, this is an inference from structure and not a direct mechanism.

### 5.3 Limitations of Docking and Molecular Dynamics Approaches

The docking and molecular dynamics approaches have their limitations, however: The binding energies and interaction geometries above are derived from either static or short‐timescale computational models whose Keap1 receptor is rigid or semi‐flexible, in the presence of implicit or simplified solvation and in the absence of explicit membrane, endosomal, or nanoparticle‐corona context. These models are not able to represent the actual intracellular concentrations, the presence of multiple thiol pools (glutathione, thioredoxin), or protein dynamics found in living MSCs, and none of the binding energies discussed herein have been tested by orthogonal biophysical methods (such as isothermal titration calorimetry, surface plasmon resonance, and cysteine adduct mass spectrometry) in an MSC‐relevant system [44]. Docking scores should therefore be viewed as only hypothesis‐generating estimates of the relative binding propensity, rather than as validated measures of intracellular reactivity.

### 5.4 Nanoparticle Surface Corona and Enhanced Keap1 Engagement

There is an important mechanistic distinction between free phytochemicals versus surface‐bound corona constituents: that the latter bind to Keap1 in a fundamentally different way than free phytochemicals [15]. The affinity of the free polyphenols for Keap1 is generally in the micromolar range, several orders of magnitude lower than the nanomolar affinity of the native ETGE degron [43]. Chemicals immobilized on the surface of a nanoparticle, however, can interact with Keap1 in a multivalent fashion, with molecular dynamics models indicating that such interactions may be one to three orders of magnitude stronger than the monovalent interactions that are possible [15]. This so far is a model‐derived prediction, not an experimentally measured value in any MSC or cell‐free biochemical system [33].

## 6 Molecular Mechanisms of Nrf2–Keap1 Activation by Phytofabricated MONPs in MSCs

**Figure 5:**
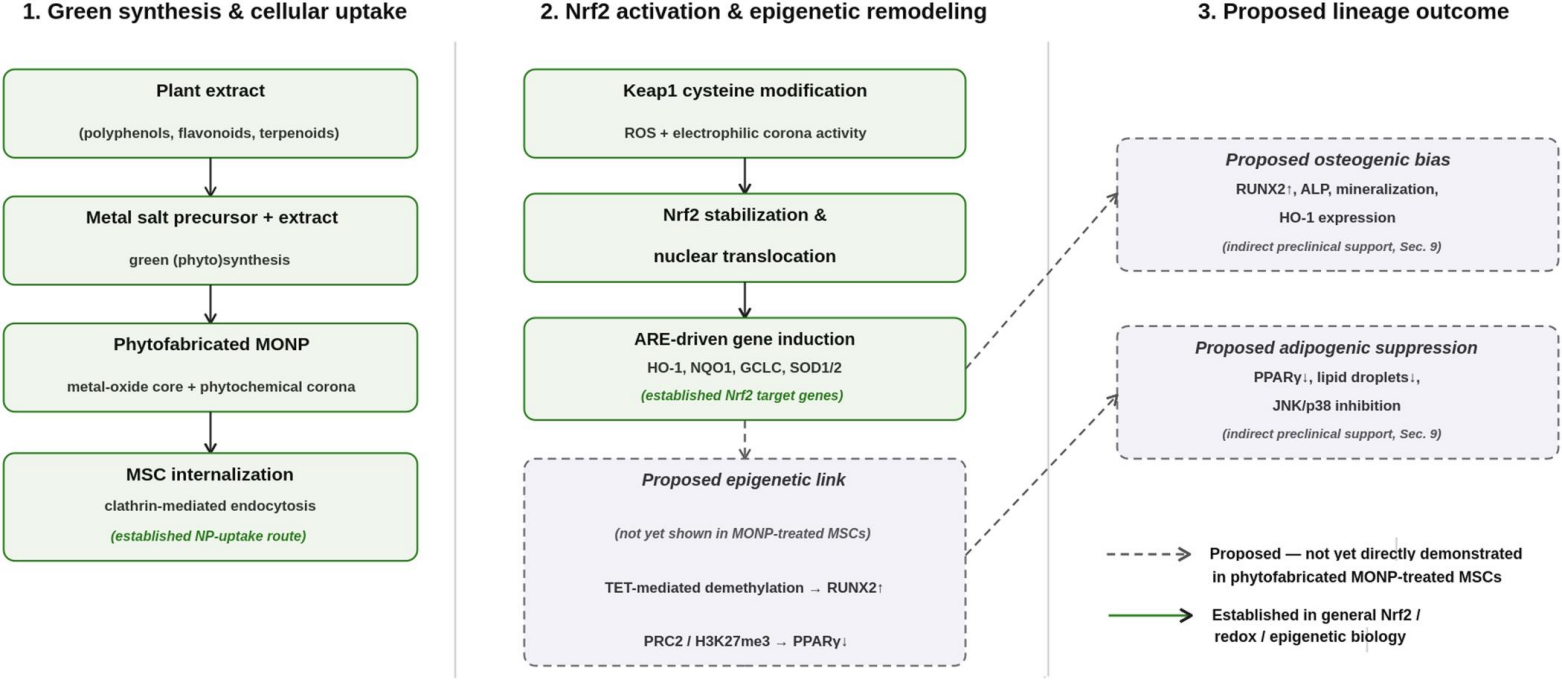
Proposed mechanistic pathway linking phytofabricated metal oxide nanoparticle (MONP) synthesis to Nrf2‐driven lineage bias in mesenchymal stem cells (Made in MS PowerPoint).

The proposed molecular trajectory of phytofabricated MONPs, from their cellular internalization to epigenetic regulation of lineage bias, is schematically presented in Figure 5.

### 6.1 Cellular Uptake and Intracellular Trafficking

The molecular mechanism of action of phytofabricated MONPs with the Nrf2–Keap1 axis is closely linked to their internalization and intracellular trafficking [45]. The moderately negative zeta potential, typical of polyphenol‐capped particles, favors uptake via clathrin‐mediated endocytosis and trafficking to the acidic late endolysosomal compartment of early endosomes. In addition, larger aggregates/corona clusters that induce membrane ruffling can also trigger macropinocytosis and caveolin‐mediated internalization that avoid the degradative process in the lysosome, leading to cargo targeting to perinuclear regions — a minor but mechanistically relevant pathway for delivering the phytochemical payload or cargo away from the degradative system towards a perinuclear location before it is lost [45].

### 6.2 Dual‐Stimulus Model: Explorable ROS Generation and Electrophilic Corona Activity

The dual‐stimulus model suggested here is different from current single‐agent Nrf2 activators, which rely on the oxidative signaling of the metal‐oxide core and the electrophilic activity of the phytochemical corona, respectively, to activate complementary components of the Keap1 cysteine‐sensing code [46]. The metal oxide core is suggested to transiently increase the amount of ROS: Surface‐catalyzed one‐electron reduction of oxygen will generate superoxide (O_2_•^−^), which can disproportionate to H_2_O_2_, and Fenton‐type reactions will catalyze the formation of hydroxyl radical (•OH) from H_2_O_2_ by the released metal ions [47]. It is suggested that the antioxidant buffering capacity of the phytochemical corona is responsible for scavenging excess radical species by hydrogen‐atom transfer, thereby maintaining the oxidative signal within a hormetic range and preventing the accumulation of cytotoxic levels of radicals [41].

### 6.3 Keap1 Cysteine Modification as the Molecular Switch

The possibility of a central switch in this activation pathway is that oxidative and electrophilic stimuli converge on complementary Keap1 cysteine residues [46]. Depending on the modifying species and cysteine sensor, two mechanistically different but potentially synergistic modification routes are proposed.

Mechanism I: Oxidative Modification of Cys273 and Cys288 in the Keap1 IVR domain by core‐derived ROS. The first step in the proposed mechanism of oxidation of these thiols is to form the sulfenic acid (–SOH) intermediate, which, in a continued oxidative stress, can be further oxidized to sulfinic acid (–SO_2_H) or can become intramolecularly paired with Cys273 and Cys288 [30]. This is predicted to cause a conformational change in the IVR that would allosterically affect the lower‐affinity DLG‐latch interaction, but initially not the higher‐affinity ETGE‐hinge interaction. This asymmetry is thought to force the Nrf2 Neh2 lysine residues out of the optimal geometry for ubiquitin transfer from the Cullin3–RBX1 complex, thereby lowering the level of polyubiquitination and allowing accumulation of Nrf2 [46].

Mechanism II is electrophilic adduct formation at Cys151 by α, β‐unsaturated carbonyl‐containing phytochemicals (such as curcuminoids, chalcones, hydroxycinnamaldehydes) liberated from the corona after endolysosomal processing. These Michael‐acceptor electrophiles are thought to form a stable thioether adduct with the Cys151 thiolate, which hinders the recruitment of Cullin3 to the surface of the BTB domain and impairs E3 ligase assembly without affecting Kelch–Nrf2 binding affinity [6].

The simultaneous engagement of both BTB‐Cys151 and IVR‐Cys273/Cys288 by a single MONP platform is hypothesized to impair Nrf2 ubiquitination through two independent structural routes simultaneously, and — if confirmed — would be expected to be more resistant to reversal by cellular thioredoxin/glutaredoxin systems than either mechanism alone. At the current moment, this dual‐mechanism synergy is not a proven phenomenon but rather a hypothesis. Such a hypothesis can be directly tested, for instance, using mass spectrometry to map the site‐specific change of cysteine residues in MSCs treated with MONP.

### 6.4 Nrf2 Stabilization, Nuclear Translocation, and are‐Driven Transcription in MSCs

Newly synthesized Nrf2 can escape from the inhibitory binding to Keap1 and sequester into the cytoplasm, where it is then imported into the nucleus by phosphorylation and binds to ARE‐containing promoters. In addition to this Keap1‐dependent pathway, a second pathway exists and regulates Nrf2 in a suppressive manner, which involves GSK‐3β‐mediated phosphorylation of Nrf2 on the Neh6 domain, stimulating its degradation by β‐TrCP. To address this pathway, phytofabricated MONPs have been proposed to inhibit this pathway by phosphorylating and inhibiting GSK‐3β at Ser9 via the PI3K/Akt‐dependent pathway, thus extending the half‐life of Nrf2 and increasing the nuclear translocation response (Table 3). Additionally, CK2‐mediated phosphorylation of Nrf2 at Thr395 and Ser433 is suggested to further facilitate its nuclear retention by inhibiting the nuclear export of Nrf2 by the Keap1 [21].

**Table 3:** Nrf2 activation mechanisms by phytofabricated MONPs and their effects on MSC lineage bias.

Activation mechanism	MONP type	Keap1/Nrf2 molecular target	Downstream ARE genes induced	Effect on osteogenic/Adipogenic balance	Reference
Oxidative Cys273/Cys288 sulfenylation	ZnO, Fe_3_O_4_	IVR DLG‐latch disruption	SOD2, TXNRD1(ThioredoxinReductase 1)	Pro‐osteogenic	[48]
Electrophilic Cys151 adduct	Curcumin‐corona MONPs	BTB–Cullin3 displacement	HO‐1, NQO1(NADPH Quinone Dehydrogenase 1)	Adipogenic suppression	[49]
PI3K/Akt‐GSK‐3β inhibition	CeO_2_, ZnO	Neh6 phosphodegron stabilization	GCLC, GCLM(Glutamate‐Cysteine Ligase Modifier Subunit)	Pro‐osteogenic	[50]
MAPK Ser40 phosphorylation	ZnO, TiO_2_	Neh2 Keap1 dissociation	NQO1(NADPH Quinone Dehydrogenase1)	Lineage shift modulation	[50]

Nrf2 in the nucleus is bound to small Maf proteins and binds to AREs in the promoter region of target genes. Within the context of the MSCs, we believe that induction of HO‐1 and NQO1 is the most consistently reported functional readout of pathway activation by Nrf2 and recommend this as the primary endpoint for confirming pathway engagement with MONP in future studies. Nrf2 also regulates GCLC and GCLM, increasing the capacity to biosynthesize glutathione and TXNRD1, strengthening the thioredoxin system, and altogether tuning the intracellular redox environment in a way that is conducive to RUNX2 transcriptional activity and osteogenic commitment [21].

## 7 Epigenetic Modulation of MSC Fate via Nrf2 and Phytofabricated Nanoparticles

### 7.1 DNA Methylation Dynamics and TET Enzyme Redox Sensitivity

At the chromatin level, lineage commitment is further secured by epigenetic changes that occur after the redox stimulus that induced a change in the cell lineage has passed [51]. The intracellular redox state is particularly sensitive to the methylation of DNA among these. The TET family dioxygenases convert 5‐methylcytosine (5mC) to 5‐hydroxymethylcytosine (5hmC), 5‐formylcytosine (5fC), and 5‐carboxylcytosine (5caC), which are intermediates that subsequently are excised via base excision repair to complete the demethylation cycle [52]. The activity of the enzyme TET is dependent on molecular oxygen, Fe^2+,^ and α‐ketoglutarate. Both increased ROS (which oxidizes Fe^2+^ to the catalytically inactive Fe^3+^ state) and disruption of the TCA cycle via oxidative degradation of α‐ketoglutarate (which limits TET‐mediated demethylation) are expected to inhibit osteogenic gene promoters and may play a role in aberrant silencing of osteogenic gene promoters [9]. The mechanism has been established in general redox biology and is the most likely direct mechanism to connect this pathway to phytofabricated MONP exposure in MSCs.

### 7.2 Histone Modification, Sirt Regulation, and Chromatin Accessibility

One other layer of epigenetic modification, histone post‐translational modification, may occur at lineage‐determining loci, and in which the activity of Nrf2 might converge with MONP‐associated phytochemicals (Table 4) [52]. In the RUNX2 locus, Nrf2 activation is suggested to lead to the recruitment of the CBP/p300 acetyltransferase complex and the resulting increase in H3K27 acetylation, which enhances chromatin accessibility and transcriptional output [9]. Under Nrf2 active conditions, it is thought that Polycomb repressive complex 2 (PRC2)‐mediated H3K27 trimethylation is maintained by repression of the enzymes that remove this repressive mark – the α‐ketoglutarate‐dependent demethylases JMJD3 and UTX – which would otherwise reopen adipogenic chromatin accessibility, thereby connecting this mechanism with the preservation of the α‐ketoglutarate pool described in section 7.1 [51]. The RUNX2/PPARγ chromatin mechanisms mentioned above are not shown directly in the context of phytofabricated‐MONP‐treated MSCs, but make sense as extensions of general epigenetic biology and should be interpreted accordingly.

**Table 4:** Epigenetic mechanisms through which Nrf2 and phytofabricated MONPs influence MSC lineage bias.

Epigenetic mechanism	Regulatory molecule involved	Nrf2‐Dependent?	Effect on osteogenic loci	Effect on adipogenic loci	Reference
TET‐mediated active DNA demethylation	TET1/TET2, Fe^2+^/α‐KG cofactors	Yes (redox buffering of TET activity)	Demethylation of RUNX2/SP7 promoters	Not directly affected	[53]
DNMT3A/3B‐mediated PPARγ promoter methylation	DNMT3A, DNMT3B	Yes (DNMT redox protection and SAM availability)	Not directly affected	Hypermethylation and silencing	[54]
SIRT1‐mediated H3K9/14 deacetylation	SIRT1, NAD^+^‐NADH‐dependent	Yes (NAD^+^ maintenance via metabolic coupling)	Permissive chromatin at RUNX2 enhancers.	Repressive	[55]
PRC2‐mediated H3K27 trimethylation	EZH2, SUZ12	Indirect (via JMJD3/UTX suppression)	Not directly affected	Silencing of PPARγ locus	[56]
HDAC/DNMT inhibition by corona phytochemicals	Quercetin, EGCG	Supportive (non‐Nrf2 direct)	Chromatin opening at osteogenic loci	DNMT‐mediated silencing of adipogenic loci.	[57]

## 8 Safety, Dose Thresholds, and Redox Toxicology of Phytofabricated MONPs in MSCs

### 8.1 The Hormesis Principle: Defining the Therapeutic Window

To make the phytofabricated MONPs truly clinically relevant platforms for lineage engineering, the dose range between redox modulation and cytotoxic oxidative damage needs to be carefully determined [58]. The biphasic dose–response curve displayed in MSC systems with ROS‐generating MONPs follows the hormesis model originally proposed by Calabrese and Baldwin: ROS exposure at low doses triggers adaptive stress‐response mechanisms, which lead to increased cellular resistance and promote osteogenesis via the induction of Nrf2–Keap1 expression [59]. When the concentration is increased beyond this hormetic level, the intracellular levels of ROS become too high to be buffered by the phytochemical corona and Nrf2‐mediated cytoprotective transcription, leading to maladaptive oxidative stress, macromolecular damage, and eventually to apoptosis or irreversible senescence [58].

### 8.2 Genotoxicity, DNA Damage, and Chromosomal Integrity

Genotoxic risk is of particular concern for the safety of phytofabricated MONPs, as genomic instability could be inherited by differentiated progeny and affect the safety of any downstream regenerative application [60]. The formation of the γH2AX (phosphorylated histone H2AX at Ser139) is a marker for DNA double‐strand breaks and has been reported in nanoparticle‐exposed MSCs. This has been correlated to the concentration of dissolved metal ions and the intracellular ROS levels in the ZnO, TiO_2,_ and Fe_3_O_4_ nanoparticle systems, and generally, the phytofabricated formulations result in lower γH2AX response than chemically synthesized formulations [60]. It is possible to distinguish the relative contributions of strand breaks and oxidative base damage using complementary quantification of oxidative base lesions, notably 8‐oxoguanine, which is produced by hydroxyl radical attack on guanine, notably by Fe_3_O_4_ dissolution [61].

### 8.3 Long‐Term Effects on Stemness, Multipotency, and Differentiation Fidelity

Transient exposure to Nrf2 retained the canonical MSC immunophenotype (CD73^+^/CD90^+^/CD105^+^, CD34^−^/CD45^−^) and trilineage differentiation capacity, suggesting that sufficiently low doses of MONP exposure would not affect multipotency. But, long‐term exposure to super‐physiological Nrf2 stimulation can, however, lead to “lineage lock” (epigenetic epigenomic locking of a chromatin state that promotes osteogenesis), as described in Section 7.2 [47, 62].

In principle, the epigenetic persistence discussed in previous sections may become a therapeutic liability rather than an advantage if the dose of H3K27 trimethylation is too high and consequently the adipogenic (and potentially chondrogenic) differentiation capacity is permanently blocked [62]. On a mechanistic basis, intermittent or pulsatile MONP exposure paradigms (transient activation of Nrf2 followed by relaxation periods) are more likely to be advantageous than exposure paradigms occupying the entire duration, as they would be expected to maintain multipotency and could introduce osteogenic bias over time [47].

## 9 Translational and Regenerative Applications

### 9.1 Bone Regeneration and Osteogenic Commitment Engineering

The ability of phytofabricated MONPs to induce osteogenic transcription factors makes them promising candidates for bone regeneration, an application area where balancing osteogenic induction, redox signaling, and inflammatory niche protection has long been a therapeutic challenge [63, 64]. In models of calvarial defect, femoral segmental defect, and ectopic bone formation, pre‐clinical studies have shown improved woven bone formation, increased trabecular connectivity, and continued RUNX2 expression with histomorphometric analyses (Table 5) [70]. In a study on ectopic bone formation, a group of MSCs was preconditioned with phytofabricated ZnO nanoparticles prior to subcutaneous implantation, which led to enhanced mineral deposition and sustained RUNX2 expression after implantation in association with cell‐based bone regeneration strategies. Nrf2‐activating interventions could be especially useful in inflammatory bone loss, such as that which occurs in osteoporosis, periodontitis‐associated alveolar bone loss, and periprosthetic osteolysis [71]. Under such circumstances, the effects of NF‐κB activation through the action of TNF‐α, IL‐1β, and RANKL together appear to inhibit osteoblast differentiation, activate osteoclasts, and maintain an oxidative stress that enhances MSC activation of PPARγ in a ROS‐dependent manner [72, 73]. Phytofabricated MONPs are suggested to interfere with this cascade at multiple sites: induction of HO‐1 leads to inhibition of NF‐κB activity through CO‐mediated suppression of IKK; restoration of glutathione levels counteracts the activity of endogenous PPARγ ligands that are lipid peroxidation products, which, in turn, recondition the osteogenic niche toward productive repair [72]. Nrf2 competent MSCs also exhibit increased phospho‐Smad1/5/8 nuclear retention and increased Id1/Id2 response to BMP‐2, suggesting the potential of using MONP preconditioning to mitigate the risks of heterotopic ossification when using high doses of BMP‐2 for spinal fusion and craniofacial reconstruction [67, 72].

**Table 5:** Preclinical purposes of Nrf2‐modulating phytofabricated MONPs in regenerative medicine.

Application model	MONP type	Target tissue	Nrf2 activation evidence	Lineage outcome	Functional endpoint	Reference
Calvarial defect model	Phytofabricated CeO_2_ NPs	Cranial bone	HO–1 immunoreactivity	Osteogenic	Bone fill percentage: osteocalcin expression	[65]
Femoral segmental defect	Phytofabricated Fe_3_O_4_ NPs	Long bone	Nrf2 nuclear translocation (IHC)	Osteogenic	Mineral apposition rate: BV/TV (μCT)	[66]
Ectopic bone formation	Phytofabricated ZnO NPs	Subcutaneous (ectopic)	RUNX2 protein elevation	Osteogenic	Alizarin Red mineralization: ALP activity	[67, 68]
Inflammatory bone loss	Phytofabricated TiO_2_ NPs	Periodontal bone	NQO1 induction; NF–κB suppression	Anti‐resorptive/Osteogenic	RANKL/OPG ratio: osteoclast count	[65]
Senescent MSC rejuvenation	Phytofabricated CeO_2_/ZnO NPs	Bone marrow	Nrf2/HO–1 restoration	Anti‐senescence/Osteogenic	p21 reduction: mineralization recovery	[69]

### 9.2 Anti‐Senescence Strategies for MSC Rejuvenation

The patient groups that would most benefit from bone regenerative therapy, such as elderly people with osteoporosis and reduced endogenous repair ability, are directly relevant to restoring the competence of Nrf2 in aged and oxidatively exhausted MSCs. Senescent MSCs exhibit increased p21/p16 expression, SASP‐mediated paracrine inflammation, and reduced ability to translocate Nrf2 into the nucleus due to hyperactivation of GSK‐3β, which may impair the responsiveness to conventional electrophilic Nrf2 activators that require intact Keap1 cysteine modification [72]. A possible solution to overcome this Keap1‐independent degradation pathway is phytofabricated MONPs that will simultaneously target Keap1 cysteine modification and the PI3K/Akt–GSK‐3β axis. In replicatively senescent bone marrow MSCs, Nrf2 restoration leads to a decrease in p21 and p16^INK4a, and to the rescue of a partial recovery of ALP activity and mineralization, and the reduction of secretion of the SASP pro‐inflammatory cytokines IL‐6/IL‐8. The phytochemical corona could also provide an extra anti‐senescence effect, by inducing SIRT1 to be activated by resveratrol and quercetin, which increased the activity of the NAD‐dependent deacetylases and helped to maintain the accessibility of chromatin at osteogenic sites [74].

### 9.3 Absence of Direct Mechanistic Evidence in MSC Systems

A major knowledge gap in this review is the lack of simultaneous characterization of Keap1 cysteine modification, Nrf2 nuclear translocation, ARE‐driven transcription, and resulting osteogenic‐versus‐adipogenic lineage outcome in MSCs treated with a defined phytofabricated MONP formulation. The evidence base is rather assembled, with three distinct literatures each exploring the effects of free phytochemicals on Nrf2 activation, the effects of MONP redox activity on non‐MSC cell types, and MSC lineage biology in response to generic oxidative stimuli, but never testing the integrated hypothesis proposed here. In our opinion, this lack of direct evidence is the single most important drawback in the current literature, and the most important experimental goal in the field going forward.

It is worth emphasizing that the broad and described antioxidant activity of phytofabricated MONPs (generalized ROS scavenging, viability maintenance, reduced inflammatory cytokines) is not comparable to site‐directed Keap1 cysteine modification in its narrow and mechanistically rigorous sense, which is required for the assignment of Nrf2 pathway activation to a particular molecular interaction. Consequently, demonstrating a downregulation of the cellular ROS or an upregulation of HO‐1 is not enough, and only direct identification of cysteine‐specific Keap1 adducts in MSCs treated with a MONP, plus pathway‐specific genetic controls (such as Keap1 cysteine‐mutant rescue) would satisfy this standard.

## 10 Conclusion

This review suggests that phytofabricated MONPs can serve as dual‐stimulus activators of the Nrf2–Keap1 axis in MSCs, simultaneously delivering metal‐core‐derived oxidative signals and electrophiles derived from the phytochemical corona to activate complementary components of the Keap1 cysteine code. If this dual targeting is confirmed experimentally, it would be a mechanistic gain over single‐stimulus activators of Nrf2, as it would break the ubiquitination of Nrf2 in two different structural ways. We also suggest that this activation of Nrf2 may serve as a redox rheostat that regulates the biased specification of MSCs, which over time may lead to metabolic reorganization (AMPK and PGC‐1α activation) and epigenetic modification (TET and SIRT1‐dependent mechanisms), to permanently commit MSCs to osteogenic fate. In this model, the corona of phytochemicals is not just a stabilizing shell, but an active player: direct interaction with Keap1, epigenetic enzyme modulation, and maintaining the pro‐osteogenic signaling at the right level while avoiding cytotoxicity — the hormetic window.

This whole system is presently based on indirect and non‐overlapping lines of evidence; this is the current field's main limitation, as detailed in Section 10. Closing this gap — through site‐specific Keap1 cysteine mapping, pathway‐specific genetic controls, and integrated dose–response studies in MSC systems — is the necessary next step before this model can inform the rational design of phytofabricated nanoplatforms for reproducible bone regeneration. More generally, if confirmed, this model could help develop a complementary approach to precisely engage a master transcriptional regulator at a specific site within the body, thereby enabling site‐directed stem cell engineering for a range of oxidative‐stress‐related pathologies where predictable progenitor cell commitment remains a clinical need and would be based on a chemically‐defined nanoparticle corona.

## Author Contributions

**Muhammad Irfan:** conceptualization, writing – review and editing, validation, supervision, methodology. **Muhammad Taha:** data curation, investigation, writing – original draft, formal analysis. **Syed Muhammad Faizan Asghar:** data curation, writing – original draft. **Muhammad Raza:** data curation, writing – original draft.

## Funding

The authors have nothing to report.

## Ethics Statement

The authors have nothing to report.

## Consent

The authors have nothing to report.

## Conflicts of Interest

The authors declare no conflicts of interest.

## Data Availability

Data sharing is not applicable to this article as no datasets were generated or analyzed during the current study.
